# Signaling Enzymes Required for Sperm Maturation and Fertilization in Mammals

**DOI:** 10.3389/fcell.2019.00341

**Published:** 2019-12-18

**Authors:** Souvik Dey, Cameron Brothag, Srinivasan Vijayaraghavan

**Affiliations:** Department of Biological Sciences, Kent State University, Kent, OH, United States

**Keywords:** hyperactivation, fertility, PP1γ2, GSK3α, epididymal sperm maturation, PP2B, PKA, contraception

## Abstract

In mammals, motility and fertilizing ability of spermatozoa develop during their passage through the epididymis. After ejaculation, sperm undergo capacitation and hyperactivation in the female reproductive tract – a motility transition that is required for sperm penetration of the egg. Both epididymal initiation of sperm motility and hyperactivation are essential for male fertility. Motility initiation in the epididymis and sperm hyperactivation involve changes in metabolism, cAMP (cyclic adenosine mono-phosphate), calcium and pH acting through protein kinases and phosphatases. Despite this knowledge, we still do not understand, in biochemical terms, how sperm acquire motility in the epididymis and how motility is altered in the female reproductive tract. Recent data show that the sperm specific protein phosphatase PP1γ2, glycogen synthase kinase 3 (GSK3), and the calcium regulated phosphatase calcineurin (PP2B), are involved in epididymal sperm maturation. The protein phosphatase PP1γ2 is present only in testis and sperm in mammals. PP1γ2 has a isoform-specific requirement for normal function of mammalian sperm. Sperm PP1γ2 is regulated by three proteins – inhibitor 2, inhibitor 3 and SDS22. Changes in phosphorylation of these three inhibitors and their binding to PP1γ2 are involved in initiation and activation of sperm motility. The inhibitors are phosphorylated by protein kinases, one of which is GSK3. The isoform GSK3α is essential for epididymal sperm maturation and fertility. Calcium levels dramatically decrease during sperm maturation and initiation of motility suggesting that the calcium activated sperm phosphatase (PP2B) activity also decreases. Loss of PP2B results in male infertility due to impaired sperm maturation in the epididymis. Thus the three signaling enzymes PP1γ2, GSK3, and PP2B along with the documented PKA (protein kinase A) have key roles in sperm maturation and hyperactivation. Significantly, all these four signaling enzymes are present as specific isoforms only in placental mammals, a testimony to their essential roles in the unique aspects of sperm function in mammals. These findings should lead to a better biochemical understanding of the basis of male infertility and should lead to novel approaches to a male contraception and managed reproduction.

## Introduction

Testicular spermatozoa in mammals are immotile and lack the ability to fertilize eggs. Motility initiation and fertilizing ability develop during their passage through the epididymis (Cooper, 1986; Bedford and Hoskins, 1990). Epididymal maturation is an absolute necessity to produce fertile spermatozoa. Sperm undergo hyperactivation in the female reproductive tract before penetration of the egg. Motile sperm failing to undergo hyperactivation cannot fertilize (Marquez and Suarez, 2008). Understanding the biochemical basis for how motility is attained and modified prior to fertilization is essential for understanding male fertility and infertility.

Sperm flagellar activity and also ciliary motility of somatic cells are known to be regulated by the intracellular mediators cAMP, calcium and pH. Capacitation is operationaly defined as functional modifications that enable sperm to fertilize eggs. Sperm undergo various changes during capacitation: (a) removal of membrane cholesterol followed leading to a decrease in cholesterol to phospholipid ratio (b) membrane hyperpolarization, rise in intra-sperm pH and cAMP levels, (c) increase in calcium uptake, and (d) an increase in protein tyrosine phosphorylation possibily due to increased protein kinase A (PKA) activity (Arcelay et al., 2008; Bailey, 2010; Alvau et al., 2016; Jin and Yang, 2017; Molina et al., 2018). It is generally accepted that these changes during capacitation lead to: (a) the ability of the sperm to bind the oocyte’s extracellular matrix, the zona pellucida (ZP) (Si and Olds-Clarke, 1999; Topper et al., 1999) and subsequently undergo the acrosome reaction, (b) hyperactivation, a whiplash flagellar motion required to penetrate the egg (Ho and Suarez, 2001), and (c) the capacity to fuse with the oocyte (Evans and Florman, 2002). Hyperactivation, however, is regulated by similar yet distinct signaling events that distinguish it from capacitation (Marquez and Suarez, 2004).

Considerable progress has been made in understanding changes in sperm cAMP, calcium, and pH during sperm activation and hyperactivation. The proteins responsible for the generation of cAMP, calcium influx and changes in pHi (intracellular pH) have been identified and their functions elucidated by biochemical and genetic approaches. Despite this information, we still do not understand, in biochemical terms, how these second messengers enable sperm to acquire motility in the epididymis and how motility is altered in the female reproductive tract. Our understanding is incomplete because other signaling enzymes can profoundly alter the action and effects of second messengers in sperm. These signaling enzymes, protein phosphatase 1 (PP1), glycogen synthase kinase 3 (GSK3) and the calcium activated phosphatase (PP2B), affect sperm function in the epididymis and in the female reproductive tract. Several reviews detail the roles of sperm cAMP-PKA and calcium metabolism (Visconti et al., 2002; Burton and McKnight, 2007; Buffone et al., 2014; Stival et al., 2016; Freitas et al., 2017; Balbach et al., 2018; Leemans et al., 2019; Stewart and Davis, 2019). Following a brief summary of the actions of cAMP-PKA, calcium and pH, the remainder of the review will be focused largely devoted to examination of the key roles of PP1, GSK3, and calcium activated protein phosphatase, PP2B (PPP3R2/CC) in mediating the actions of the second messengers during sperm maturation and fertilization.

## Roles of cAmp and Protein Kinase a in Sperm Function

Motility can be induced in demembranated testicular and caput epididymal sperm in the presence of ATP and cAMP, as well as appropriate calcium levels and pH (Lindemann, 1978; Mohri and Yanagimachi, 1980; Yeung, 1984; Lindemann et al., 1987; Bedford and Hoskins, 1990; Lesich et al., 2008). Early studies on the motility effects of phosphodiesterase inhibitors (such as caffeine, theophylline, and IBMX), on intact sperm led to the discovery of the role of cAMP in sperm motility regulation (Dougherty et al., 1976; Jiang et al., 1984). Phosphodiesterase inhibitors or cell permeable cAMP analogs can initiate motility in immotile spermatozoa and stimulate increased motility in motile sperm. Motility activation of demembranated sperm is abrogated in the presence of protein phosphatases in the reactivation medium, suggesting that cAMP mediates its motility effect through protein phosphorylation (Takahashi et al., 1985; Murofushi et al., 1986). An elevation of intra-sperm cAMP is also thought to be involved in motility initiation in the epididymis (Hoskins et al., 1974; Amann et al., 1982; Vijayaraghavan et al., 1985). A unique, hormone insensitive but bicarbonate sensitive, soluble adenylyl cyclase (sAC) is responsible for the synthesis of cAMP in sperm. Sperm cAMP levels should also be regulated by phosphodiesterases (PDEs) enzymes that degrade cAMP. Of the eleven PDEs, PDE1, PDE4, PDE8, PDE10, and PDE11 are present in testis and sperm (Omori and Kotera, 2007; Keravis and Lugnier, 2012).

It is well known that cAMP acts through a protein kinase (PKA). Knockout of the sperm sAC or PKA leads to infertility due to impaired sperm motility and the inability of sperm to undergo hyperactivation (Hess et al., 2005; Xie et al., 2006). Mature sperm contain PKA which is composed of regulatory subunit RIIα and a sperm specific catalytic subunit Cα2. Targeted knock-out of sperm-specific Cα2 results in male infertility. Spermatogenesis in these mutant mice is normal, while kinetic vigor and beat amplitude of epididymal sperm were markedly reduced. Mutant sperm are unable to undergo bicarbonate induced motility stimulation, and hyperactivation. It is known that PKA in cells owes it specificity and function to its localization through anchoring proteins known as AKAPs (Colledge and Scott, 1999). Sperm contain at least two PKA anchoring proteins AKAP 3 and 4 (also known as AKAP110 and 82, respectively) (Lin et al., 1995; Vijayaraghavan et al., 1999). Targeted deletion of AKAP 3 or 4 results in sperm dysfunction and male infertility (Jackson Labs Mouse Repository) (Miki et al., 2002). AKAP82 has been shown to play significant roles mediating the PKA action in murine and human sperm (Moss et al., 1999; Turner et al., 1999).

One of the downstream effects of PKA has been suggested to involve activation of protein tyrosine kinases (PTKs): proline−rich tyrosine kinase 2 (PYK2), ABL (Abelson murine leukemia viral oncogene homolog 1), SRC, and FER (Baker et al., 2006, 2009; Alvau et al., 2016; Brukman et al., 2019). However, targeted disruption of these PTKs did not impair male fertility. Thus the exact role, of protein tyrosine phosphorylation in sperm function is still not well understood. PKA has also been suggested to regulate cAMP-phosphodiesterase activity. Phosphorylation by PKA has been shown to increase catalytic activities of PDE4 and PDE11 (Sette and Conti, 1996; Yuasa et al., 2000) suggesting a feedback regulation of sperm cAMP levels. Hyper-activated motility in sperm capacitation may also include phospholipase D-dependent actin polymerization (Itach et al., 2012). Thus while it is known that cAMP and PKA are essential in sperm the exact downstream biochemical actions of PKA are still unknown.

## The Roles of pHi, Calcium and Catsper Channels in Sperm Function

**FIGURE 1:**
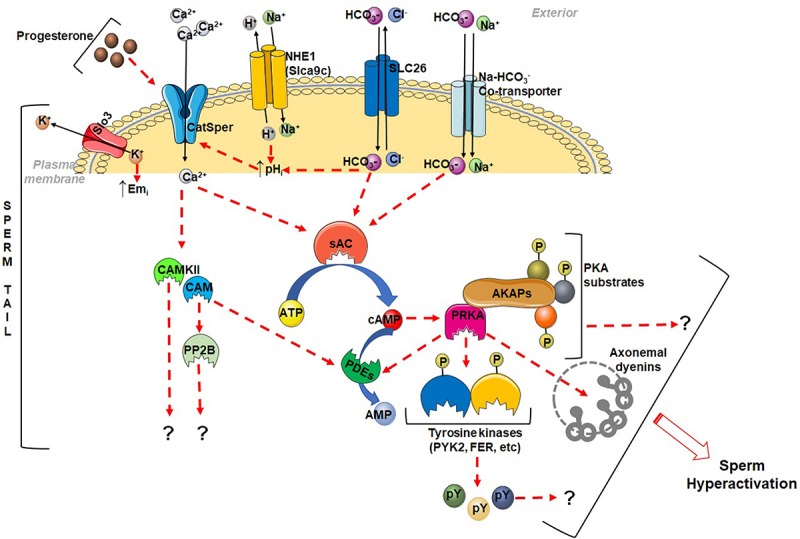
Schematic diagram showing some of the signaling events during sperm hyperactivation. CatSper channels are activated either by progesterone or alkalization of sperm cytosol to permit calcium entry. The Cl^–^–HCO_3_^–^ exchanger, SLC26A3 and the Na^+^–HCO_3_^–^ co-transporter, and Na^+^–H^+^ exchanger, NHE1 (Slca9c), aid in alkalization of sperm pHi. Slo3 K^+^-channels are activated by alkalization and causes hyperpolarization. Both Ca^2+^ and HCO_3_^–^ stimulate sperm sAC to increase cAMP levels. Cyclic AMP, in turn activates sperm PKA (PRKA), which is expected to phosphorylate several substrates including protein tyrosine kinases (PTKs). Ca^2+^ also activates calmodulin (CAM) and calmodulin dependent phosphatase, calcineurin (PP2B) and a protein kinase CAMKII. The downstream effect and mechanism of action of these enzymes in promoting sperm fertilization is not well understood. Index: dotted arrow indicates activation, upward arrow denotes increase, and question mark (?) indicates unknown mechanism.

The role of pH in regulating flagellar motion was first recognized in the mechanism underlying activation of sea urchin sperm in sea water, which occurs due to increased pHi mediated by a sodium – proton exchange (Lee et al., 1983). Several studies have now shown that increasing intracellular pH activates motility of sperm in a number of species (Vijayaraghavan et al., 1985; Hamamah and Gatti, 1998; Carr and Acott, 2005; Nishigaki et al., 2014). A change in pHi is also thought to accompany sperm maturation in the epididymis (Vijayaraghavan et al., 1985). Acidic pH and the high concentration of lactate which acts as a membrane permeable proton carrier render sperm immotile in the luminal fluid of the cauda epididymis (Carr et al., 1985; Vijayaraghavan and Hoskins, 1988). Dilution of the quiescent caudal sperm, during ejaculation or in a buffer *in vitro*, results in initiation of vigorous motility. More recently, elevation of pHi has also been recognized to be a key event required for initiation of calcium influx during sperm hyperactivation. Knockout of channels, Slo3, NHE1 (Slca9c) or SLC26A3, responsible for changes in pH renders sperm infertile (Wang et al., 2007; Chen et al., 2009; Santi et al., 2010; Zeng et al., 2011; Chavez et al., 2014; Toure, 2019). A significant advance in understanding hyperactivation came from the discovery of calcium channels (CatSper) as essential mediators of sperm calcium influx. The calcium channel is composed of several essential subunits (Qi et al., 2007; Chang and Suarez, 2011; Singh and Rajender, 2015; Williams et al., 2015; Sun et al., 2017). Alkalinization of the sperm cytosol followed by activation of calcium channels (CatSper) triggers hyperactivation (Carlson et al., 2003; Qi et al., 2007; Lishko and Kirichok, 2010; Chung et al., 2011; Chavez et al., 2014; Lishko and Mannowetz, 2018; Orta et al., 2018). Prevention of the increase in pHi or loss of CatSper leads to infertility. A recent study shows that CatSper channels also mediate Zn^2+^-dependent stimulation of sperm hyperactivation (Allouche-Fitoussi et al., 2018). A model for sperm hyperactivation incorporating the functions of cAMP-PKA, calcium, and pHi is shown in Figure 1. This model is based on those presented in reviews noted earlier on the mechanisms regulating sperm function. Absent in theses models are the essential functions of the signaling enzymes PP1γ2, GSK3α, and PP2B required for sperm motility initiation and fertility.

## Protein Phosphatase Isoform, Pp1γ2 in Sperm Function

**FIGURE 2:**
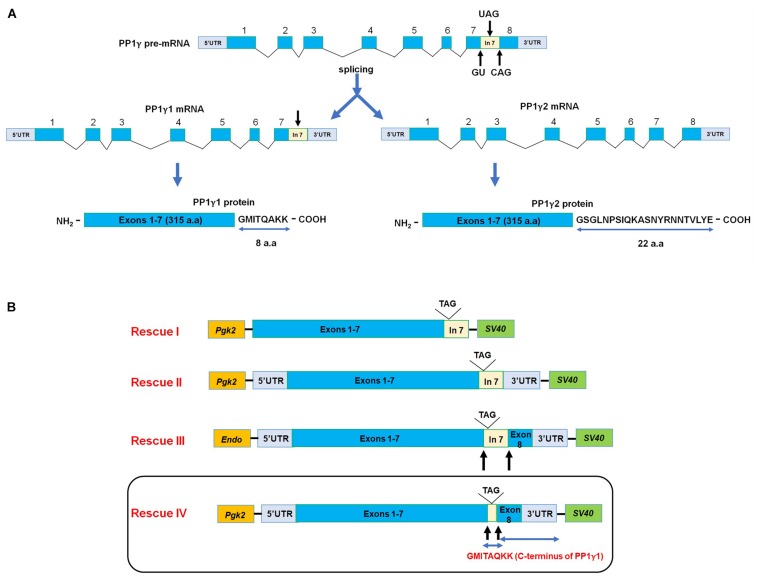
**(A)** Generation of PP1γ isoforms. The *Ppp1cc* contains 8 exons and 7 introns. The Pp1γ1 mature mRNA (2.3 kb) contains exons 1 through 7. Intron 7 is retained as an extended exon leading to the eight amino acid C-terminus of PP1γ1 (note that “exon 8” is part of its 3′UTR). The PP1γ1 encodes a protein containing 323 amino acids derived from the seven exons along with the 8 amino acid C-terminus from the extended exon 7. In the post-meiotic germ cells in testis the intron 7 is spliced out, thus, producing a shorter Pp1γ2 transcript of approximately 1.7 kb. Exon 8 codes for the 22 amino acid C-terminus in PP1γ2. Thus, the amino acid sequences of PP1γ1 and PP1γ2 are identical in all respects except for their extreme C-termini. **(B)** Constructs for generating transgenic PP1γ1 mice. Rescues I–III constructs contain the entire or a portion of intron 7 which is part of the 3′UTR of the messenger RNA for PP1γ1. There was little or no transgenic expression of PP1γ1 in testis of mice generated from these constructs. The last rescue construct (Rescue IV) lacks the 0.9 kb region of intron 7 following the stop codon in PP1γ1 mRNA. Transcript from this construct will resemble PP1γ2 mRNA except that PP1γ1 protein will be produced. The transgenic mice produced from this construct expressed high testis levels of transgenic PP1γ1 and rescued spermatogenesis but not sperm fertility.

The presence of a protein kinase in a cell requires a corresponding protein phosphatase. The phosphorylation status of a protein is the result of the opposing activities of protein kinases and protein phosphatases. Based on the observation that protein phosphatases prevent motility initiation of demembranated spermatozoa and also enzyme activity measurements in sperm extracts (Swarup and Garbers, 1982; Takahashi et al., 1985; Murofushi et al., 1986), it was long suspected that a protein phosphatase regulates flagellar motility. However, the identity of the phosphatase and details of its regulation were not known. Research on protein phosphatases was boosted by the discovery of compounds, calyculin A, okadaic acid, and microcystin, isolated from marine organisms that are potent inhibitors of protein phosphatases (Cohen, 1990; Cohen et al., 1990; Fernandez et al., 2002). The inhibitors display distinct inhibition profiles against the serine/threonine phosphatases PP1, PP2A, and PP2B enabling their identification in cellular extracts (da Cruz e Silva et al., 1995). The protein phosphatase inhibitors, calyculin A and okadaic acid, were among the most potent in initiating and stimulating motility of sperm (Smith et al., 1996, 1999; Vijayaraghavan et al., 1996). The inhibitors initiated and stimulated epididymal sperm motility at nanomolar and micromolar concentrations, respectively. The inhibition profile of enzyme activity in sperm extracts suggested that the predominant phosphatase in sperm was protein phosphatase 1 (PP1) along with measurable levels of PP2A. There are four isoforms of PP1, PP1α, PP1β, PP1γ1, and PP1γ2, encoded by three genes (Okano et al., 1997; Lin et al., 1999). The amino acid sequences of all four proteins are essentially identical except at their C-termini. The two PP1 isoforms, PP1γ1 and PP1γ2, are alternate transcripts from a single gene, *Ppp1c* (Figure 2A). Based on enzyme activity profiles and western blot analysis, we found that the predominant serine/threonine protein phosphatase in spermatozoa is PP1γ2. High PP1γ2 activity is associated with low sperm motility, while low PP1γ2 activity is associated with vigorous motility (Smith et al., 1996, 1999; Vijayaraghavan et al., 1996). A decline in PP1γ2 activity occurs during epididymal sperm maturation, due to a decrease in its catalytic activity. Other laboratories have shown that the phosphatase inhibitors also promote hyperactivated sperm motility and acrosome reaction (Furuya et al., 1992a, b; Signorelli et al., 2013; Rotfeld et al., 2014; Matsuura and Yogo, 2015; Tsirulnikov et al., 2019). The enzyme PP1γ2 is present in spermatozoa of a wide range of mammalian species including human and non-human primates (Chakrabarti et al., 2007a; Vijayaraghavan et al., 2007).

In most tissues and cells, the loss of any one of the PP1 isoforms is compensated by one of the other isoforms. In yeast the loss of its endogenous protein phosphatase (GLC7) can be functionally replaced by one of the four mammalian PP1 isoforms, highlighting their functional equivalence (Gibbons et al., 2007). Because PP1γ2 was implicated in sperm motility it was of interest to see how its loss would affect sperm function. Loss of *Ppp1cc* leads to defects in spermiogenesis and lack of sperm in the epididymis (Varmuza et al., 1999; Chakrabarti et al., 2007b). The enzyme PP1γ2 has a dual role, one, during spermatogenesis and the other in sperm after their exit from the seminiferous tubules. This dual role of PP1γ2 is intriguing because loss of the enzymes of cAMP metabolism and action, sAC or PKA in testis, does not impair sperm morphogenesis or sperm formation. It is likely that other serine/threonine protein kinases along with PP1γ2 are responsible for regulation of protein phosphorylation during spermatogenesis (Kawa et al., 2006; Xu et al., 2007; MacLeod et al., 2014; Cruz et al., 2019). Due to its requirement in spermatogenesis, it is not possible to obtain sperm lacking PP1γ2, which is a limitation for the study of the enzyme in mature sperm.

The only phenotype resulting from the knockout of *Ppp1cc* is male infertility. Females lacking *Ppp1cc* are normal and fertile (Table 1). Conditional knockout in post-meiotic developing germ cells also has the same phenotype as the global loss of *Ppp1cc* showing the requirement of *Ppp1cc* only in differentiating germ cells in testis (Sinha et al., 2013). It should be noted that the *Ppp1cc* gene is responsible for expression of both the PP1 isoforms: PP1γ1 and PP1γ2. Since, PP1γ2 is the predominant isoform in testis, it strongly suggests, but does not prove that the reason for male infertility in mice lacking *Ppp1cc* is likely due to the absence of only PP1γ2 in differentiating spermatogenic cells. It was later confirmed that, despite the global absence of PP1γ1, transgenic expression of PP1γ2 driven by the PGK2 promoter in spermatocytes and spermatids of *Ppp1cc* null mice restored spermatogenesis, sperm function, and fertility (Sinha et al., 2012). These data provide compelling evidence that the PP1γ2 isoform expressed only in developing germ cells is sufficient for normal sperm function and fertility.

**TABLE 1:** Fertility status of knockout mouse lines.

**Group**	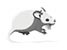 **Mouse lines** 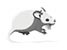	**Fertility status (female)**	**Fertility status (male)**
1	PP1 (*Ppp1cc*) global knockout (Chakrabarti et al., 2007b)	Fertile	Infertile
	PP1γ2 conditional (testis-specific) knockout (Sinha et al., 2013)	Fertile	Infertile
	PP1γ1 rescue (in *Ppp1cc* knockout background) (Dudiki et al., 2019)	Fertile	Sub-fertile
2	GSK3α global knockout (Bhattacharjee et al., 2015)	Fertile	Infertile
	GSK3α conditional (testis-specific) knockout (Bhattacharjee et al., 2018)	Fertile	Infertile
3	GSK3β global knockout (Hoeflich et al., 2000)	Embryonically lethal	
	GSK3β conditional (testis-specific) knockout (Bhattacharjee et al., 2018)	Fertile	Fertile
4	PKACα2 (testis isoform specific) knockout (Nolan et al., 2004)	Fertile	Infertile
5	sAC (*Adcy10*) knockout (Hess et al., 2005; Xie et al., 2006)	Fertile	Infertile
6	PPP3R2 (sperm isoform specific) knockout (Miyata et al., 2015)	Fertile	Infertile
	PPP3CC (sperm isoform specific) knockout (Miyata et al., 2015)	Fertile	Infertile

The phosphatase isoform, PP1γ2, is present only in eutherian mammals. Sperm from non-mammalian species and invertebrates contain one of the three PP1 isoforms – PP1α, PP1β, or PP1γ1 – which is able to support sperm motility and fertility in these species. The fact that PP1γ2 alone is sufficient for male fertility does not necessarily suggest that the other PP1 isoforms would be unable to functionally replace PP1γ2. Is the requirement for PP1γ2 an evolutionary accident or is there an isoform specific function for it in mammalian sperm? Can expressing the PP1γ1 isoform in testis, restore spermatogenesis and fertility of *Ppp1cc* null mice? Employing the same strategy used in the PP1γ2 transgenic rescue approach, the PGK2 promoter was used to drive transgenic expression of PP1γ1. This approach which was successful for PP1γ2 expression (Sinha et al., 2012), failed to transgenically express PP1γ1 (Dudiki et al., 2019) in testis. The transgene construct in all these failed attempts contained portions of the intron preceding exon 8, which is part of the 3′UTR of the mRNA for PP1γ1 (Figure 2B). In the fourth attempt, removal of this entire portion of the 3′UTR in the cDNA in the transgene construct (Figure 2B) led to robust expression of PP1γ1 in developing spermatocytes and spermatids (Dudiki et al., 2019). Transgenic expression of PP1γ1 in testis of *Ppp1cc* null mice was able to fully restore spermatogenesis. However, sperm function and fertility were severely compromised in these PP1γ1 rescue mice. Motility of PP1γ1-bearing sperm was diminished and their flagellar beat amplitude was severely dampened (Dudiki et al., 2019). Fertility defects in the rescue mice were most likely due to the inability of sperm bearing PP1γ1 to undergo hyperactivation. Thus PP1γ2 is essential in sperm for its normal function and fertility.

**FIGURE 3:**
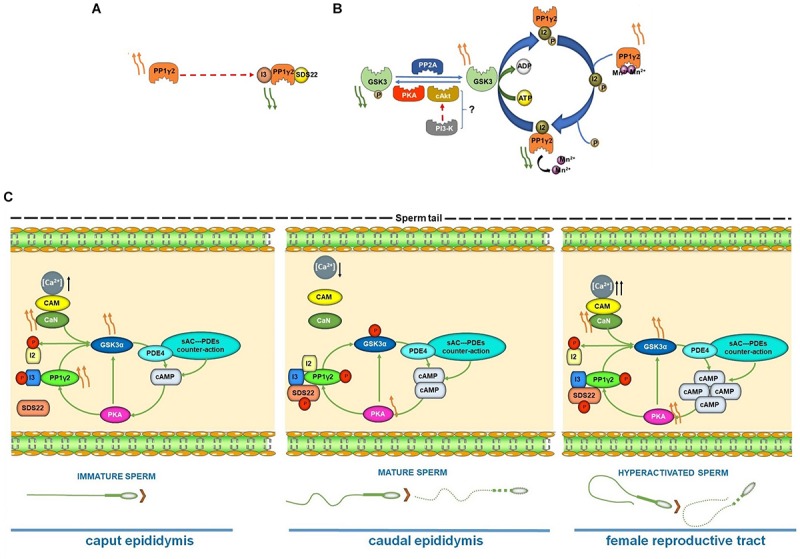
**(A–B)** Regulation of PP1γ2 activity by I3, SDS22 and I2. **(A)** I3 and SDS bound PP1γ2 is catalytically inactive. Binding of these inhibitors to PP1γ2 is altered in caudal compared to caput sperm and in sperm from infertile transgenic KO mice (see text). **(B)** Inhibitor 2, a regulator of PP1γ2, is known to be phosphorylated by GSK3. In caput but not in caudal epididymal sperm I2 is expected to be phosphorylated due to changes in GSK3 activity. This is one of the ways by which catalytic activity of PP1γ2 is thought to decrease during epididymal sperm maturation. **(C)** Schematic diagram of the proposed interrelationship between GSK3α, PKA Cα2, PP1γ2 and PP2B (PPP3R2/CC) during epididymal sperm maturation and sperm hyperactivation in female reproductive tract. Curved orange arrow(s) indicate relative degree of activities of the enzymes; straight black arrow(s) denote relative level of the ion/protein inside the cell.

A specific isoform requirement of a protein is usually thought to arise due to isoform specific binding partners for that protein or due to its unique biochemical activity. An isoform specific function could also be due to the restricted spatio-temporal expression of the protein isoform during cell or tissue development. Despite the knowledge that PP1γ2 is the only PP1 isoform expressed in developing spermatocytes and spermatids (Chakrabarti et al., 2007b). it was anticipated that specific binding proteins for PP1γ2 exist in testis and sperm. However, binding partners of sperm PP1γ2 identified so far are ubiquitous in tissues and organisms and are known to bind to all PP1 isoforms (Goswami et al., 2019). That is, these proteins would bind to any PP1 isoform if present in sperm, just as they do in other cells and tissues. The three protein regulators of PP1γ2 identified are PPP1R2 (inhibitor I2), PPP1R7 (SDS22), and PPP1R11 (inhibitor I3). These three regulators/inhibitors are evolutionarily ancient and conserved across species (Heroes et al., 2013), play key roles in mitosis and other cellular functions (Peggie et al., 2002; Wang et al., 2008; Eiteneuer et al., 2014). Thus, loss of any one them is likely to cause embryonic lethality. It is intriguing that the sperm specific isoform, PP1γ2, is regulated by these ancient, ubiquitous, and essential PP1 binding proteins. The three regulators share localization with PP1γ2 in the head and the principal piece of sperm. The association of inhibitors to PP1γ2 changes during epididymal sperm maturation. In immotile caput epididymal sperm, PPP1R2 and PPP1R7 are not bound to PP1γ2, whereas in motile caudal sperm, all three inhibitors are bound as hetero-dimers or hetero-trimers (Goswami et al., 2019) (Table 2 and Figures 3A,B). In caudal sperm from male mice lacking sAC and GSK3 (see below), where motility and fertility are impaired, the association of PP1γ2 to the inhibitors resembles immature caput sperm. In sperm containing PP1γ1 the association of these inhibitors are altered resembling that of PP1γ2 in immotile caput epididymal sperm (Goswami et al., 2019) (Table 2). It is known that binding of inhibitor 2 to PP1 is regulated by GSK3 (Figure 3B). It is likely that binding of the other two inhibitors, PPP1R7 and PPP1R2, to PP1γ2 are also regulated by phosphorylation. Changes in the associations of the regulators with PP1γ2, are likely part of biochemical mechanisms responsible for the development of motility and fertilizing ability of sperm.

**TABLE 2:** The binding profile of the regulators with PP1γ2 in caput and caudal epididymal sperm is summarized in the table along with data with caudal sperm from Gsk3α knockout, PKA Cα2 knockout and sAC knockout mice where the binding status of sds22 resembles that in wild type caput sperm.

**Group**	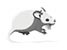 **Mouse lines**		**I2**	**SDS22**	**I3**
1	Wild type (Goswami et al., 2019)	Caput	Not bound	Not bound	Bound
		Caudal	Bound	Bound	Bound
2	GSK3α global knockout (Goswami et al., 2019)	Caput	Not bound	Not bound	Bound
		Caudal	Bound	Not bound	Bound
3	PKACα2 (testis isoform specific) knockout (Goswami, 2018)	Caput	Not bound	Not bound	Bound
		Caudal	Bound	Not bound	Bound
4	sAC (*Adcy10*) knockout (Goswami et al., 2019)	Caput	Not bound	Not bound	Bound
		Caudal	Bound	Not bound	Bound

*Thus, epididymal sperm maturation appears to be altered in these knockout mice.*

A recently identified potential binding protein for PP1γ2 is CCDC181 (Schwarz et al., 2017). It is likely that CCDC181 binds to PP1γ1 and PP1γ2 with differing affinities leading to preferential localization of PP1γ2 to the flagellum. Determination of the protein targets of PP1γ2 in the flagellum and how CCDC181 regulates PP1γ2 is under active investigation.

## Glycogen Synthase Kinase 3α, Gsk3α, in Sperm

The enzyme GSK3, a serine/threonine protein kinase, was named GSK-3 because it was discovered after PKA and phosphorylase kinase (GSK-1 and GSK-2). Two other GS kinases, GSK-4 and -5, named based on their relative elution profiles in phospho-cellulose chromatography of muscle extracts, were later renamed as casein kinase 1 and 2, respectively (Parker et al., 1982). The enzyme GSK3, retained its original name even though it was later found to be a key signaling component of a large number of cellular processes (Kaidanovich-Beilin and Woodgett, 2011; Medina and Wandosell, 2011). An array of functions attributed to GSK3 include insulin action, regulation of cell survival, apoptosis, embryonic development, Wnt/β-catenin and hedgehog signaling, and growth factor action. It is also a target for drug development in several clinical disorders including cancer (Jope et al., 2007).

In mammals, GSK3 is ubiquitous and is expressed as two isoforms, GSK3α and GSK3β, encoded by different genes. The catalytic domains of the two isoforms are 98% identical while their N- and C-termini are distinctive (Woodgett, 1990). While there are reports ascribing distinct roles for each of the isoforms (McNeill and Woodgett, 2010) under most circumstances the two isoforms are redundant and functionally interchangeable. Knockout of *Gsk3*β in mice causes late embryonic lethality (Hoeflich et al., 2000). The inability of GSK3α to substitute for GSK3β in the developing embryo may be due to the non-overlapping expression of the two isoforms. Conditional knockout of the floxed *Gsk3*β alleles on a *Gsk3*α null background show that complete loss of both isoforms impairs signaling and tissue function. However, one allele of *Gsk3*β or *Gsk3*α on a *Gsk3*α or *Gsk3*β null background, respectively, is sufficient to maintain normal Wnt signaling and tissue function (Doble et al., 2007; McNeill and Woodgett, 2010) highlighting the functional redundancy of the two isoforms in most tissues and cell types.

The protein, GSK3 was first discovered as an enzyme responsible for activation of PP1γ2 in bovine sperm (Vijayaraghavan et al., 1996). Both α and β isoforms of GSK3 are present in sperm. Immotile caput sperm contain four-fold higher GSK3 activity than motile caudal epididymal sperm. Both tyrosine phosphorylation (which stimulates catalytic activity) and serine phosphorylation of GSK3 (an inhibitory mechanism) increase significantly in sperm during their passage through the epididymis (Somanath et al., 2004). Incubation of motile or immotile sperm with compounds that activate PKA (e.g., dbcAMP) or inhibit protein phosphatase (e.g., calyculin A) is accompanied by increases in GSK3 serine phosphorylation and motility stimulation. GSK3 tyrosine phosphorylation which is believed to be autoregulatory, remains unchanged during capacitation, while only GSK3α ser21 phosphorylation is altered during this event (Dey et al., 2019a, b).

It was recently shown that *Gsk3*α null mice exhibit male infertility (Bhattacharjee et al., 2015, 2018). Knockout of GSK3α in post-meiotic testicular germ cells, using the Cre-Lox strategy, also results in male infertility. Mice with a testis knockout of GSK3β are normal and fertile. Thus, GSK3α has an isoform specific function in sperm. Analysis of sperm lacking GSK3α showed that adenine nucleotide levels, energy metabolism, and protein phosphatase and kinase activities were affected suggesting impaired sperm maturation in the epididymis. A recent report also documents the role for GSK3 and a non-canonical Wnt signaling during epididymal sperm maturation: loss of Wnt signaling in sperm results in male infertility (Koch et al., 2015). The activity of GSK3α isoform has also been correlated with human sperm motility (Freitas et al., 2019). The inability of GSK3β to replace GSK3α, only in testis and sperm, is surprising given the fact that the two isoforms are functionally interchangeable in most cellular contexts and in tissues. Thus, despite the presence of both GSK3 isoforms, mammalian sperm are unique in their requirement for the GSK3α isoform. Taken together, these studies, support the notion that GSK3α is essential for epididymal sperm maturation, motility, and fertilization.

## Calcineurin in Sperm Function

Calcineurin (also known as PP2B or PPP3C) is a serine/threonine phosphatase regulated by calcium. In response to an elevation of cellular calcium, calmodulin binds to a calmodulin binding region of the catalytic subunit PPP3C. This binding causes an auto-inhibitory arm of calcineurin to move away from the substrate binding site thus activating the enzyme by enabling its access to substrates (Rusnak and Mertz, 2000; Parra and Rothermel, 2017). The catalytic activity of the enzyme is also regulated by calcium binding to a regulatory subunit (PPP3R2). Regulation and function of calcineurin in several cell types has been extensively studied (Rusnak and Mertz, 2000; Parra and Rothermel, 2017). More than two decades ago a role for a calcium regulated protein phosphatase was proposed in the regulation of sperm motility (Tash et al., 1988; Tash and Bracho, 1994). In non-mammalian sperm, calcineurin has been shown to have role in activation of progressive motility and egg activation (Levasseur et al., 2013; Krapf et al., 2014). The catalytic and regulatory subunits of calcineurin are present as testis-specific isoforms, PPP3CC and PPP3R2. It was shown by super resolution microscopy that the catalytic subunit of calcineurin, PPP3CC, is localized near the quadrilateral structures of CatSper in the axoneme (Chung et al., 2014). In CatSper1-deficient spermatozoa, PPP3CC can be seen localized mostly to the axoneme but disappears from the quadrilateral structures. Another report showed that pharmacological inhibition of calmodulin affects protein tyrosine phosphorylation seen during sperm capacitation (Navarrete et al., 2015). In another study, micromolar amounts of FK506 has been demonstrated to prevent sperm acrosomal exocytosis (Castillo Bennett et al., 2010). A recent report now shows that knockout of either *Ppp3CC* or *Ppp3R2* present only in testis and sperm resulted in male infertility (Miyata et al., 2015). Sperm numbers and testis weights in these knockout mice are normal; but sperm motility is impaired with a stiffened mid-piece. The *Ppp3CC* or *Ppp3R2* knockout mice are infertile *in vivo*. Sperm from these knockout mice also cannot fertilize eggs *in vitro*. Surprisingly, wild type sperm treated with the calcineurin inhibitors, FK506 and cyclosporine, did not affect *in vitro* fertilization. Thus, infertility was thought to be due to impaired sperm function in the male reproductive tract. Calcineurin inhibitors injected into mice resulted in reversible male infertility. The investigators concluded that calcineurin was required for epididymal sperm maturation: genetic disruption or pharmacological inhibition *in vivo* affected sperm maturation causing infertility (Miyata et al., 2015).

The questions of how calcineurin may act and how it is activated during epididymal sperm maturation were not addressed. Recent data show that calcineurin and GSK3 are interrelated in their roles in epididymal sperm maturation and absence of calcineurin increases GSK3 phosphorylation resulting in its lower catalytic activity (Dey et al., 2019b). It is suspected that calcineurin regulates mitochondrial energization directly and glycolysis indirectly through its effect on GSK3. It is likely that high calcium levels in immature sperm (Vijayaraghavan and Hoskins, 1989, 1990) activates calcineurin.

## Interrelationship Between Pka, Pp1, Gsk3α, and Pp2B

As described earlier the requirement and the roles of cAMP and the kinase activated by it, PKA, in sperm are well known. The relationship between PKA and GSK3 in sperm was indicated by the fact that cAMP analogs increased GSK3-α/β phosphorylation, without any isoform specificity. Phosphorylation of both GSK3 α and β isoforms were reduced and its catalytic activity increased in sperm with diminished cAMP (using KH7, a sAC inhibitor), or due to knock out of sAC. That is, GSK3 is a target of PKA phosphorylation. This relationship between GSK3 and PKA was further validated in GSK3α knockout mice. Loss of GSK3α or β by targeted disruption or pharmacological inhibition of the enzyme significantly reduced sperm cAMP levels (Dey et al., 2018). The decrease in cAMP levels was attributed to increased phosphodiesterase activity. Together these data support the possibility that GSK3 and cAMP form an interrelated regulatory loop (Dey et al., 2018).

Inhibition of the predominant sperm protein phosphatase PP1γ2 by calyculin A significantly increased phosphorylation of both GSK3 isoforms (Ser21/9) in caudal epididymal sperm and a concomitant decrease in its catalytic activity. Conversely, increased GSK3 activity is associated with increased PP1γ2 activity. One of the ways by which GSK3 regulates PP1 activity is by its phosphorylation of the inhibitor I2 as discussed earlier. Phosphorylated I2 dissociates from PP1 leading to its activation (Figure 3B). The roles of binding and dissociation of PP1γ2 binding proteins due to their reversible phosphorylation is a feature of the regulation of the phosphatase in sperm (Goswami et al., 2019). Sperm PP2A also targets GSK3, without any isoform specificity; however, its role in regulating GSK3 has not been investigated in detail (Dudiki et al., 2015).

New information shows that not only is GSK3 a target of PP1γ2, but it is also regulated by calcineurin. While PP1γ2 acts on both isoforms of GSK3 (Somanath et al., 2004), calcineurin preferentially dephosphorylates only the GSK3α isoform (Dey et al., 2019a, b). Phosphorylation of the GSK3α (Ser21) isoform is elevated in sperm lacking calcineurin (Dey et al., 2019a, b). Following a decrease in their catalytic activities in caudal epididymal sperm during motility initiation, surprisingly the catalytic activities of both GSK3α and calcineurin increase during sperm capacitation and hyperactivation, recapitulating the situation in caput sperm (Figure 3C) (Dey et al., 2019a, b). Increased calcineurin activity, presumably following increased sperm Ca^2+^ during hyperactivation is responsible for decreased phosphorylation and increased catalytic activity of GSK3α. Pharmacologic inhibition of calcineurin during capacitation abrogated this decrease in phosphorylation and the increase in GSK3 activity. Increased activities of GSK3α and PP2B appear to be characteristics of not only capacitated sperm, but also, paradoxically, of immature caput epididymal sperm. Thus, phosphatases (PP1γ2 and PP2B) and the kinases (PKA and GSK3) are mechanistically interrelated during epididymal initiation of motility and also during fertilization of the egg (summarized in Figure 3C).

## The Proteins Pp1γ2, Gsk3α, Pka cα2, and Ppp3R2/Cc Present Only in Sperm Are Conserved in Mammals

**FIGURE 4:**
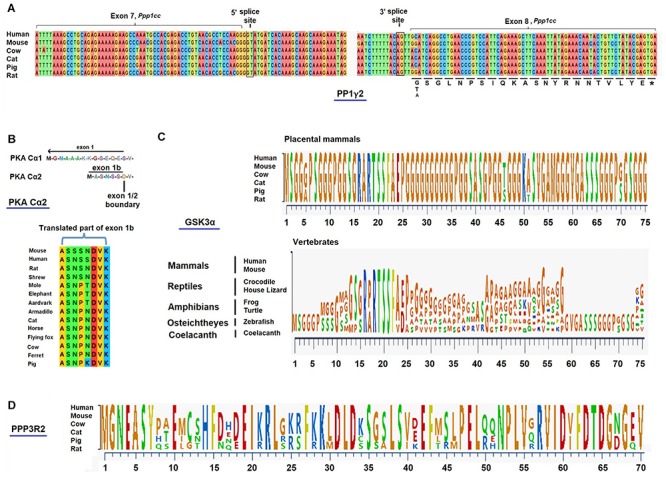
**(A)** Conservation of C-terminus of PPP1CC2 in mammals. The 5′ and 3′ splice sites for the generation of PP1γ2 in *Ppp1cc* is found only in mammals. These splice sites and a recognizable exon 8 are absent in *Ppp1cc* in the genomes of the duck billed platypus and the non-mammalian vertebrates. The C-terminal 22 amino acid residues (316–337) are virtually identical in all placental mammals for which annotated genomic data bases exist. Sequences from a few of these mammals are shown above. **(B)** PKA catalytic subunit in somatic cells (Cα1) and sperm (Cα2) are derived from alternate exons 1a and 1b. Cα1 is present in somatic cell. Cα2 produced in testis using exon 1b is present only in mammals. Exon 1b is present and conserved in all placental mammals. **(C)** Conservation of GSK3α isoform in mammals. The glycine rich N-terminus of the GSK3 alpha isoform is conserved in placental mammalian species, but not in non-mammalian vertebrates. **(D)** Conservation of testis-specific isoform of PP2B, PPP3R2 (regulatory subunit) across different mammalian species. While the entire PPP3R2 is present and conserved in all mammals only a portion of the N-terminus sequence of PPP3R2 is shown.

As noted earlier the PP1γ2 isoform in sperm, is present only in mammals. Examination of the available annotated genome databases shows that monotremes and other non-mammalian vertebrates contain the *Ppp1cc* gene but the PP1γ2 isoform cannot form due to the absence of splice sites at exon 7 in the gene. Also absent is a region corresponding to exon 8, which is highly conserved and present in the *Ppp1cc* in all mammals (Figure 4A). This PP1γ2 isoform derived from exon 8 has a unique 22 amino acid C-terminus present and conserved in all mammals (Figure 4A). The somatic isoform, PP1γ1 has a six amino acid C-terminus derived from the extended exon 7. Aside from these differences in the C-termini, the rest of 315 amino acid sequences of the both PP1γ1 and PP1γ2 are identical. Why mammalian sperm contain only PP1γ2 is unknown.

It is known that knockout of the enzyme synthesizing cAMP in sperm, sAC, and that of PKA result in male infertility. It is intriguing that the catalytic subunit of PKA is also expressed as a sperm specific isoform (PrkaCA also known as PKA Cα2) with a unique six amino acid N-terminus due to expression of an alternate exon, exon 1b (Agustin et al., 2000; Desseyn et al., 2000; San Agustin and Witman, 2001) (Figure 4B). The exon 1a codes for 12 amino acids of the N-terminus of the ubiquitous and the somatic cell version of the catalytic subunit. Aside from this difference in their N-termini the primary sequences of the rest of the catalytic subunits are identical. The reason this different N-terminus is required in testis and sperm remains puzzling because both sperm and somatic cell forms of the PKA catalytic subunits have identical biochemical properties (Vetter et al., 2011). Removal of exon 1b leading to the loss of PKA in sperm, renders males infertile (Nolan et al., 2004). Whether replacement of the sperm form of the enzyme with the somatic form of the PKA catalytic subunit would sustain normal sperm function is not known. However, it is intriguing that exon 1b, the sperm specific isoform of PKA, is present only in mammals (Soberg et al., 2017). Non-mammalian species only contain the isoform derived from utilization of exon 1a (Figure 4B).

A germ cell–enriched protein, viz. sperm PKA interacting factor (SPIF), was found to be co-expressed and co-regulated with PKACα2 and with t-complex protein (TCP)-11 (Stanger et al., 2016). These three proteins constitute part of a novel trimeric complex in murine spermatozoa. During capacitation, the SPIF undergoes phosphorylation leading to a molecular rearrangement that brings PKACα2 and TCP11 into close proximity of each other. These results could explain how PKA Cα2 functions as a specific isoform complexed with SPIF and TCP11 during capacitation and fertilization.

The GSK3α isoform is essential in mammalian sperm despite the fact both GSK3α and β isoforms are interchangeable in other cells and tissues. GSK3α arose in vertebrates presumably by gene duplication of GSK3β which is the only isoform in invertebrates. Among vertebrates the GSK3α isoform is absent in birds (Alon et al., 2011). Sequence comparison of the extended glycine rich N-terminus present only in GSK3α shows that this sequence segment is highly conserved only in placental mammals. This extended N-terminus is present, but not conserved in non-mammalian vertebrates (Figure 4C). We predict that sperm from non-mammalian vertebrates contain only GSK3β and not GSK3α. In fact, we have shown that Xenopus sperm contains only GSK3β despite the fact both GSK3 isoforms are present in the genome of this species. These observations are compatible with the possibility that mammalian sperm contain a GSK3α-specific binding protein. Efforts to identify a isoform-specific binding protein using a two-hybrid approach with testis cDNA yielded a number of GSK3 binding proteins, but these GSK3 interactors were not isoform specific (Freitas et al., 2019). However, a yeast two-hybrid approach using human fetal cDNA yielded four GSK3α-specific binding proteins one of which appears to play a role in regulating circadian rhythm (Zeidner et al., 2011). One of these four GSK3α binding proteins is CENPV which is highly expressed in testis (NCBI, mouse ENCODE transcriptome data). CENPV also binds to tubulin (Honda et al., 2009) and is therefore, expected to be localized along the length of the flagellum. Thus, GSK3α in the flagellum is likely to orchestrate phosphorylation of proteins involved in regulating sperm motility and hyperactivation. The requirement of sperm GSK3α with its conserved of N-terminus suggests a role for it in maturation and fertilization events unique to mammals.

A relatively recent addition to the list of signaling enzymes regulating sperm function is the calcium regulated phosphatase, PP2B. Catalytic activity of PP2B is required in sperm during their passage through the epididymis as is the case with GSK3α. Sperm and testis express specific isoforms of the catalytic and regulatory calcineurin, PPP3CC and PPP3R2, respectively. Examination of the genomic sequences of several species shows that PPP3R2 is present only in eutherian mammalians and its amino acid sequence is remarkably conserved in the 121 mammals for which annotated genomic databases exist (Figure 4D).

**FIGURE 5:**
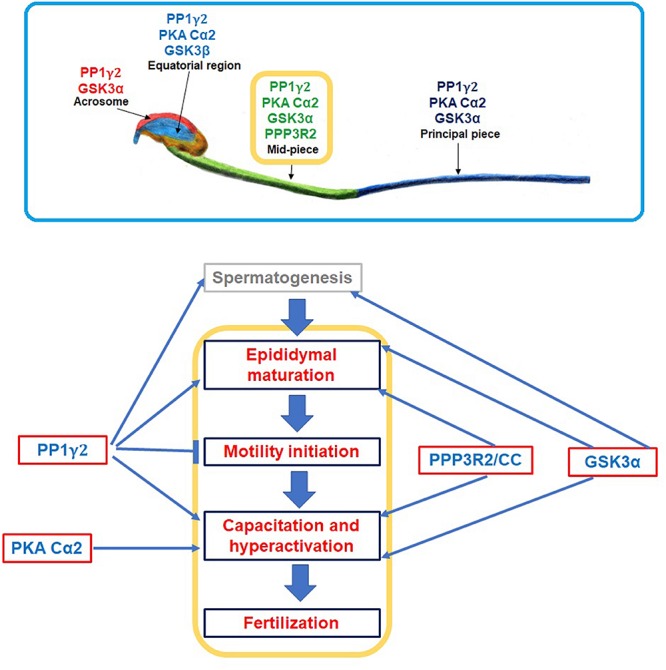
A simplified summary of spatial and temporal organization of GSK3α, PKA Cα2, PP1γ2, and PP2B (PPP3R2/CC). Upper panel image was adopted and modified from Dey et al. (2018, 2019a); it shows localization of these enzymes in different compartments of a sperm cell; all of these four enzyme isoforms co-localizes only in sperm midpiece. Lower panel demonstrates how these enzymes regulate sperm functions starting from its synthesis (i.e., spermatogenesis) till fertilization. (↑) and (⊤) indicate stimulatory and inhibitory effect, respectively.

Several predominantly testis-expressed proteins present in mammalian sperm have been identified as mammal-specific. These include proteins involved in DNA binding, sperm egg binding or those required for required for the unique structural features of mammalian sperm, such as protamine 3, SMCP, and ADAM proteins (Cho, 2012; Luis Villanueva-Canas et al., 2017). However, the observation that the four signaling enzymes suggested to be mechanistically interrelated are mammal specific isoforms is significant, suggesting a unique function in male gametes (Figure 3C). The specific isoforms PP1γ2, GSK3, PKA and PP2B play key roles in regulation sperm motility and hyperactivation, a phenomenon unique only to mammals (Figure 5). It would appear that their roles in these physiological functions in mammals arise due to their location in the flagellum. Figure 5 shows the intrasperm localization of these enzymes. It would be interesting to determine if one or more of CatSper complexes located along the flagellum are mammal specific. The two possible binding proteins for PP1γ2 in the flagellum are CCDC181 (Schwarz et al., 2017) and PPP1R32 (Cifuentes et al., 2018), both of which are expressed predominantly or exclusively in the testis. As described above, the GSK3α binding protein CENPV should be present bound to the flagellum because CENPV also binds to tubulin, which in sperm is only present in tail (Honda et al., 2009). Sperm calcineurin has been shown by high resolution microscopy to be localized along the flagellum (Chung et al., 2014). The nature of the binding protein for calcineurin in sperm is not known. Finally, PKA is known to bind AKAP3, which is present along the sperm flagellum (Vijayaraghavan et al., 1999). How the unique N-terminus of the sperm specific PKA catalytic subunit affects this localization is not known. Thus, all the four signaling proteins localized in the flagellum (PP1γ2, GSK3α, PP2B, and PKA) are likely to determine the phosphorylation status of proteins orchestrating motility and metabolism required for normal sperm function (Figure 3C).

In summary, considerable data show that the enzymes PP1γ2, GSK3 and PP2B, along with PKA, are mechanistically interrelated in regulating the two physiological processes unique to mammals: epididymal sperm maturation and sperm hyperactivation preceding fertilization (Figure 5).

## Author Contributions

SV and SD contributed to the conception and outline of the review. SD and CB organized the database. SV wrote the first draft of the manuscript. SD wrote the sections of the manuscript. All authors contributed to the manuscript reading and revisions.

## Conflict of Interest

The authors declare that the research was conducted in the absence of any commercial or financial relationships that could be construed as a potential conflict of interest.
